# Supplementary material for: Investigating the Impact of Tillage and Crop Rotation on the Prevalence of phlD-Carrying Pseudomonas Potentially Involved in Disease Suppression
Source: Microorganisms. 2023 Sep 30;11(10):2459. doi: 10.3390/microorganisms11102459 (PMC10609274; doi:10.3390/microorganisms11102459)
Supplement: Supplementary file 1 [file microorganisms-11-02459-s001.zip › microorganisms-2566562-supplementary.pdf]

microorganisms

Supplementary materials

Table S1. Crop rotation history under conventional and conservation tillage practices.

Year	Conventional tillage (CT)					Conservation strip tillage (ST)				
**2012-13**	**Wheat**	**Barley**	**Oats**	**OSR**	**Wheat**	**Wheat**	**Barley**	Oats	OSR	Wheat
2013-14	Barley	OSR	Wheat	Wheat	Wheat	Barley	OSR	Wheat	Wheat	Wheat
**2014-15**	**OSR**	**Wheat**	Barley	Oats	**Wheat**	**OSR**	**Wheat**	Barley	Oats	**Wheat**
**2015-16**	**Wheat**	Oats	**OSR**	Wheat	**Wheat**	**Wheat**	Oats	**OSR**	Wheat	**Wheat**

*Samples collected in year 2014-15 and 2015-16 were highted with orange colour. The text highlighted with bold letters describe the OSR and wheat samples collected for analysis in each year and show previous crop history. .

Table S2. Crop management practices used for the wheat and OSR winter crops.

Crops Name	Variety	Sowing date	Harvest date	Seed rate Seeds /m²	Fertilizer application kg/ha N	P	K	Fungicide application	Herbicide application	Insecticide application
**OSR**	**Compass**	**2/9/14**	**27/7/15**	**Year *14/15* 50**	**225**	**25.2**	108	Proline 0.4 L/ha (in year 2014/15) & 0.6 L/ha (in year 205/16) for Light leaf spot, Filan 0.5 kg/ha for Phoma stem canker	Katamaran 2L/ha (Pre-sowing), Falcon 1L/ha (Post-sowing)	Slug pellets 5kg /ha (in year 204/15) & 4 kg/ha (in year 205/16) Sumi Alpha for Flea Beetle
		9/9/15	25/7/16	Year *15/16* 60						
Wheat (R) Wheat (M)	J B Diego	30/9/14	25/8/15	300	225	25.2	108	Caldrum **+** Bravo (2.5 +1 L/ha), Adexar + Bravo (1.6 + 1 L/ha), Prosaro 1 L/ha for Septoria disease	Roundup 0.4 L/ha (Pre-sowing), Alister 1 L/ha (Post-sowing), Pacifica 0.5 kg/ha	Sumi Alpha for Aphids
		30/9/15	27/8/16							

*(R) means wheat in rotation where the previous crop was OSR and (M) means continuously growing wheat or wheat monoculture where previous crop was wheat.

Table S3. Physical and chemical characterisation of the soil substrates.

Mineral content (g·kg^{-1})	Rotation CT	Rotation ST	Monoculture CT	Monoculture ST
Total carbon	3.83	3.87	3.72	3.75
Total nitrogen	0.27	0.27	0.26	0.26
Soil organic matter	6.70	6.82	6.67	6.69
Soil organic carbon	2.74	2.77	2.71	2.71
C/N ratio	12.87	13.12	12.45	12.46
pH	6.52	7.12	6.80	7.05
Soil classification	Sandy clay loam			

*Rotation = OSR and wheat crops were grown in rotation. Monoculture = wheat was continuosly grown in the same plot under CT and ST practices.

Table S4. Climate and weather conditions during the experiment.

Months	Max air temp ^{0}C		Min air temp ^{0}C		Mean air temp ^{0}C	Total rainfall (mm)	Mean wind Speed (ms^{-1})	Max wind speed (ms^{-1})	Solar radiation (Wm^{-2})	Humidity (%)	CBL pressure (Pa)	Soil temperature ^{0}C at			
	High	Low	Low	High								5cm	10cm	20cm	30cm
**2014/15**															
September	22.8	15.6	4.4	14	14.3	0.61	2.18	07.6	1146	82.05	1013	16.1	16.1	15.9	15.6
October	17.7	13.2	0.5	14.6	11.4	4.46	4.41	12.5	0613	85.14	1001	11.4	11.6	11.9	11.9

November	14.2	4.2	-2.1	8.1	7.2	5.52	3.02	09.5	0300	90.49	0996	7.2	7.5	8.1	8.3
December	13.5	3.0	-5.3	10.8	5.6	1.54	4.32	11.7	0230	87.94	1011	4.5	4.8	5.4	5.5
January	16.2	2.1	-4.8	8.5	4.99	2.13	5.33	14.3	0285	85.05	1004	4.0	4.1	4.7	4.8
February	12.8	3.3	-5.6	7.2	4.30	1.30	3.99	11.7	0480	86.54	1009	4.0	4.1	4.3	4.3
March	14.2	5.6	-2.2	9.2	6.24	1.73	4.76	13.5	1005	80.05	1012	6.5	6.5	6.5	6.4
April	18.9	9.6	-0.4	7.2	8.65	0.88	3.48	10.4	1638	77.59	1014	10.9	10.9	10.5	10.1
May	19.2	9.6	1.6	11.2	10.21	2.88	4.54	12.5	1629	79.04	1006	12.6	12.5	12.1	11.8
June	24.0	11.9	3.6	15.7	13.43	0.99	4.04	10.9	2029	74.85	1012	17.4	17.2	16.4	15.9
July	23.4	16.5	6.4	13.8	14.58	2.56	4.04	11.4	1485	80.19	1005	16.7	16.6	16.5	16.1
August	23.0	14.8	6.0	13.9	14.64	2.68	3.57	10.9	1408	80.02	1005	16.7	16.6	16.4	16.0
**2015/16**															
September	19.4	13.3	3.8	13.3	12.43	0.92	3.15	09.1	0993	81.53	1010	13.7	13.7	13.8	13.6
October	19.1	11.1	-0.3	12.3	10.21	1.83	2.93	09.3	0523	87.17	1010	10.9	11.0	11.2	11.2
November	17.7	5.6	-2.1	13.9	9.21	3.67	5.05	15.2	0255	86.56	1005	8.8	8.9	9.4	9.4
December	14.1	6.3	-0.1	11.7	8.57	8.74	6.22	17.5	0146	89.45	1001	7.7	7.8	8.1	8.2
January	13.6	4.9	-3.1	11.9	5.91	3.58	4.74	13.5	0230	90.77	0996	5.2	5.3	5.7	5.8
February	13.1	5.1	-3.2	7.8	4.85	3.30	4.49	12.7	0440	87.28	1001	4.8	4.9	5.4	5.4
March	14.1	5.5	-3.5	7.4	6.13	1.31	3.77	10.9	0862	83.28	1007	6.5	6.5	6.6	6.5
April	17.2	7.4	-1.4	7.0	7.51	2.14	3.74	11.7	1323	79.48	1005	9.3	9.2	9.0	8.7
May	21.9	11.6	3.4	12.4	12.36	1.99	3.26	9.74	1742.57	80.65	1007	15.3	15.0	14.2	13.7
June	23.7	14.3	4.7	15.1	15.07	2.06	3.21	9.52	1604.07	83.67	1007	18.1	17.8	17.4	17.0
July	27.5	15.3	6.8	15.4	15.99	0.95	3.63	10.41	1515.66	83.31	1008	18.7	18.5	18.0	17.6
August	23.5	15.9	7.8	15.4	16.03	1.48	3.86	10.55	1295.70	82.10	1010	17.9	17.8	17.6	17.3

*Parameters were recorded for example; temperature in degree Celsius, rainfall in millimetres, wind speed in meters per second, humidity in percentage, Convective Boundary Layer (CBL) atmospheric pressure in Pascal (Pa), and solar radiation in Watt per square meter. OSR and wheat crops were sawn in month of September and harvested in July and August respectively. Maximum and minimum air temperatures were reported from the range of high to low.

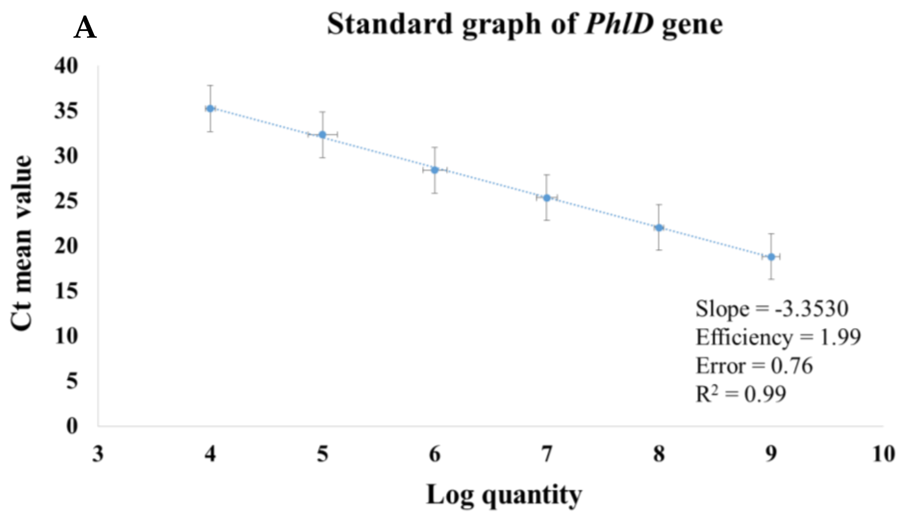

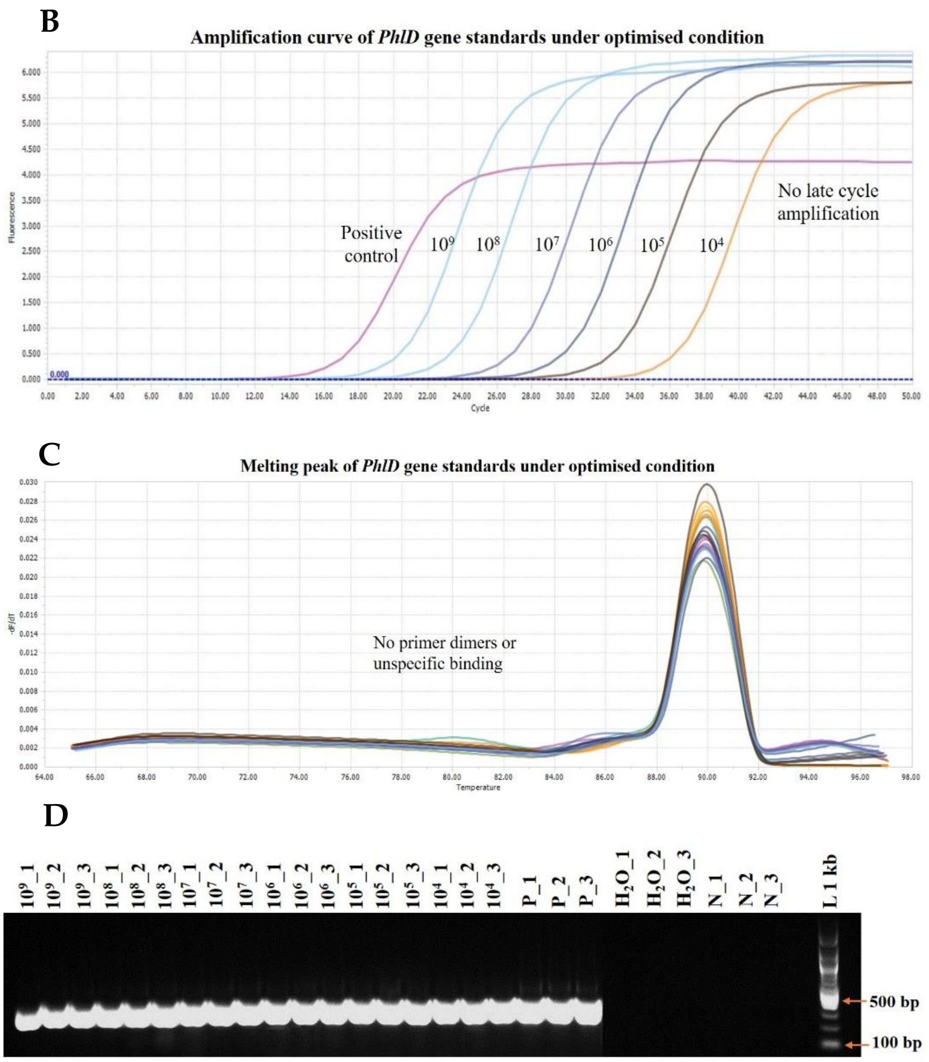

Figure S1. Standard curve was generated using 10-fold dilution of the bacterial DNA from *P. fluorescens* strain F113. Each dilution was analysed in triplicate. **A**, Standard curve with the Ct plotted against the log of the starting quantity of DNA for each dilution. **B**, Amplification curves of the dilution series. **C**, Melting pick of each dilution D, PCR amplification on agarose gel of each dilution, positive, negative and water control in triplicate. (**P**) positive control *P. fluorescens* F113 DNA, (**N**) negative control *E.coli* DNA.

Table S5. Ct value and associated calculations for *PhlD* gene copy number per gram of rhizosphere soil sample from year 2014/15.

Growth Stage	Crop type	Tillage type	Mean Ct value	Esti-mated log value	Copy number	Soil in g	DNA con. per µl	Total DNA con. in 50 µl	Total copy no.	Copy no in g soil	Log value g soil^{-1}
Vegeta-tive	OSR	CT	34.343	4.307	20261	0.25	81.3	4065	8235917	32943668	7.52
Vegeta-tive	OSR	CT	35.567	3.938	8674	0.25	86.0	4300	3730032	14920128	7.17
Vegeta-tive	OSR	CT	33.527	4.553	35694	0.25	83.0	4150	14812863	59251450	7.77
Vegeta-tive	OSR	ST	29.420	5.789	615623	0.25	89.0	4450	273952454	1095809816	9.04
Vegeta-tive	OSR	ST	27.783	6.282	1915129	0.25	95.0	4750	909686199	3638744796	9.56
Vegeta-tive	OSR	ST	30.280	5.530	339098	0.25	91.0	4550	154289809	617159236	8.79

Vegetative	Wheat	CT	31.157	5.266	184636	0.25	76.7	3835	70808061	283232243	8.45
Vegetative	Wheat	CT	31.403	5.192	155609	0.25	84.3	4215	65589021	262356083	8.42
Vegetative	Wheat	CT	31.733	5.093	123781	0.25	80.8	4040	50007518	200030073	8.30
Vegetative	Wheat	ST	26.757	6.591	3902825	0.25	89.7	4485	1750416971	7001667885	9.85
Vegetative	Wheat	ST	30.373	5.502	317847	0.25	92.3	4615	146686511	586746046	8.77
Vegetative	Wheat	ST	28.680	6.012	1028410	0.25	90.4	4520	464841518	1859366072	9.27
Vegetative	Wheat (C)	CT	34.713	4.195	15676	0.25	95.8	4790	7508640	30034560	7.48
Vegetative	Wheat (C)	CT	33.293	4.623	41962	0.25	88.7	4435	18610282	74441127	7.87
Vegetative	Wheat (C)	CT	32.527	4.854	71407	0.25	86.2	4310	30776453	123105811	8.09
Vegetative	Wheat (C)	ST	30.493	5.466	292469	0.25	96.7	4835	141408953	565635811	8.75
Vegetative	Wheat (C)	ST	30.650	5.419	262362	0.25	96.0	4800	125933547	503734188	8.70
Vegetative	Wheat (C)	ST	29.537	5.754	567781	0.25	91.5	4575	259759877	1039039507	9.02
Flowering	OSR	CT	37.547	3.342	2198	0.25	72.4	3620	795564	3182254	6.50
Flowering	OSR	CT	36.507	3.655	4520	0.25	77.3	3865	1747078	6988311	6.84
Flowering	OSR	CT	37.623	3.319	2084	0.25	74.0	3700	771045	3084181	6.49
Flowering	OSR	ST	33.587	4.535	34239	0.25	100	5000	17119532	68478126	7.84
Flowering	OSR	ST	34.360	4.302	20028	0.25	94.6	4730	9473127	37892509	7.58
Flowering	OSR	ST	32.143	4.969	93150	0.25	97.0	4850	45177689	180710757	8.26
Flowering	Wheat	CT	36.477	3.664	4615	0.25	78.1	3905	1802263	7209054	6.86
Flowering	Wheat	CT	35.323	4.012	10269	0.25	84.9	4245	4359149	17436596	7.24
Flowering	Wheat	CT	35.850	3.853	7127	0.25	87.3	4365	3111017	12444068	7.09
Flowering	Wheat	ST	34.490	4.262	18301	0.25	85.7	4285	7842120	31368482	7.50
Flowering	Wheat	ST	31.697	5.104	126969	0.25	83.9	4195	53263302	213053207	8.33
Flowering	Wheat	ST	36.407	3.685	4845	0.25	91.3	4565	2211661	8846644	6.95
Flowering	Wheat (C)	CT	37.423	3.379	2394	0.25	79.2	3960	947989	3791957	6.58
Flowering	Wheat (C)	CT	35.683	3.903	8000	0.25	81.7	4085	3268150	13072599	7.12
Flowering	Wheat (C)	CT	37.367	3.396	2490	0.25	70.9	3545	882652	3530609	6.55
Flowering	Wheat (C)	ST	31.367	5.203	159616	0.25	99.9	4995	79728068	318912273	8.50
Flowering	Wheat (C)	ST	35.787	3.872	7447	0.25	93.7	4685	3488997	13955988	7.14
Flowering	Wheat (C)	ST	33.587	4.535	34239	0.25	87.6	4380	14996710	59986839	7.78

Harvesting	OSR	CT	36.507	3.655	4520	0.25	89.3	4465	2018293	8073171	6.91
Harvesting	OSR	CT	39.510	2.751	563	0.25	73.0	3650	205589	822357	5.92
Harvesting	OSR	CT	32.730	4.793	62017	0.25	84.0	4200	26046938	104187750	8.02
Harvesting	OSR	ST	28.453	6.080	1203448	0.25	82.1	4105	494015293	1976061172	9.30
Harvesting	OSR	ST	27.523	6.360	2293491	0.25	88.0	4400	1009136176	4036544705	9.61
Harvesting	OSR	ST	26.690	6.611	4087481	0.25	78.0	3900	1594117642	6376470567	9.80
Harvesting	Wheat	CT	ND	ND	ND	0.25	70.2	3510	ND	ND	ND
Harvesting	Wheat	CT	ND	ND	ND	0.25	75.9	3795	ND	ND	ND
Harvesting	Wheat	CT	ND	ND	ND	0.25	77.7	3885	ND	ND	ND
Harvesting	Wheat	ST	35.690	3.901	7963	0.25	81.1	4055	3229186	12916744	7.11
Harvesting	Wheat	ST	37.157	3.459	2880	0.25	80.0	4000	1152060	4608238	6.66
Harvesting	Wheat	ST	38.847	2.950	892	0.25	85.5	4275	381423	1525692	6.18
Harvesting	Wheat (C)	CT	39.810	2.660	457	0.25	88.3	4415	201973	807892	5.91
Harvesting	Wheat (C)	CT	40.307	2.511	324	0.25	80.6	4030	130646	522584	5.72
Harvesting	Wheat (C)	CT	41.420	2.176	150	0.25	86.0	4300	64414	257655	5.41
Harvesting	Wheat (C)	ST	36.440	3.675	4734	0.25	81.0	4050	1917319	7669277	6.88
Harvesting	Wheat (C)	ST	37.260	3.428	2681	0.25	89.0	4450	1193043	4772172	6.68
Harvesting	Wheat (C)	ST	39.293	2.816	655	0.25	86.0	4300	281466	1125863	6.05

Table S6. Ct value and associated calculations for *PhlD* gene copy number per gram of rhizosphere soil sample from year 2015/16.

Growth Stage	Tillage type	Crop type	Mean Ct value	Estimated log value	Copy number	Soil in g	DNA con. per µl	Total DNA con. in 50 µl	Total copy no.	Copy no in g soil	Log value g soil^{-1}
Vegetative	OSR	CT	33.287	4.625	42157	0.25	86.0	4300	18127397	72509588	7.86
Vegetative	OSR	CT	35.687	3.902	7982	0.25	80.6	4030	3216704	12866816	7.11
Vegetative	OSR	CT	35.377	3.995	9896	0.25	88.3	4415	4369114	17476456	7.24
Vegetative	OSR	ST	29.533	5.755	569095	0.25	86.0	4300	244710870	978843481	8.99
Vegetative	OSR	ST	27.693	6.309	2038457	0.25	89.0	4450	907113317	3628453269	9.56
Vegetative	OSR	ST	30.437	5.483	304190	0.25	81.0	4050	123197144	492788575	8.69
Vegetative	Wheat	CT	34.400	4.290	19480	0.25	85.5	4275	8327648	33310590	7.52
Vegetative	Wheat	CT	33.600	4.531	33924	0.25	80.0	4000	13569584	54278335	7.73
Vegetative	Wheat	CT	33.437	4.580	37992	0.25	81.8	4090	15538809	62155238	7.79
Vegetative	Wheat	ST	27.517	6.363	2304118	0.25	77.7	3885	895149949	3580599797	9.55
Vegetative	Wheat	ST	31.663	5.114	129937	0.25	75.9	3795	49311277	197245106	8.30
Vegetative	Wheat	ST	28.307	6.125	1332281	0.25	70.2	3510	467630763	1870523050	9.27
Vegetative	Wheat (C)	CT	35.467	3.968	9297	0.25	78.0	3900	3625966	14503864	7.16

Vegetative	Wheat (C)	CT	35.577	3.935	8615	0.25	88.0	4400	3790402	15161608	7.18
Vegetative	Wheat (C)	CT	34.330	4.311	20449	0.25	82.1	4105	8394212	33576846	7.53
Vegetative	Wheat (C)	ST	30.090	5.588	386852	0.25	84.0	4200	162477680	649910721	8.81
Vegetative	Wheat (C)	ST	29.427	5.787	612784	0.25	73.0	3650	223666210	894664841	8.95
Vegetative	Wheat (C)	ST	30.427	5.486	306307	0.25	89.3	4465	136766138	547064552	8.74
Flowering	OSR	CT	41.177	2.249	177	0.25	87.6	4380	77672	310688	5.49
Flowering	OSR	CT	40.273	2.521	332	0.25	93.7	4685	155432	621726	5.79
Flowering	OSR	CT	39.450	2.769	587	0.25	99.9	4995	293300	1173200	6.07
Flowering	OSR	ST	36.633	3.617	4140	0.25	70.9	3545	1467686	5870745	6.77
Flowering	OSR	ST	39.470	2.763	579	0.25	81.7	4085	236562	946250	5.98
Flowering	OSR	ST	37.617	3.321	2094	0.25	79.2	3960	829050	3316202	6.52
Flowering	Wheat	CT	47.467	0.355	2	0.25	90.4	4520	1023	4090	3.61
Flowering	Wheat	CT	45.480	0.953	9	0.25	92.3	4615	4140	16560	4.22
Flowering	Wheat	CT	48.553	0.027	1	0.25	89.7	4485	478	1910	3.28
Flowering	Wheat	ST	33.360	4.603	40067	0.25	87.3	4365	17489077	69956306	7.84
Flowering	Wheat	ST	31.500	5.163	145520	0.25	84.9	4245	61773194	247092776	8.39
Flowering	Wheat	ST	35.150	4.064	11580	0.25	78.1	3905	4522145	18088579	7.26
Flowering	Wheat (C)	CT	45.500	0.947	9	0.25	90.4	4520	3999	15996	4.20
Flowering	Wheat (C)	CT	48.200	0.134	1	0.25	69.4	3468	472	1887	3.28
Flowering	Wheat (C)	CT	48.630	0.004	1	0.25	70.1	3506	354	1416	3.15
Flowering	Wheat (C)	ST	40.450	2.468	294	0.25	74.0	3700	108600	434398	5.64
Flowering	Wheat (C)	ST	47.730	0.275	2	0.25	77.3	3865	728	2914	3.46
Flowering	Wheat (C)	ST	48.497	0.044	1	0.25	72.4	3620	401	1604	3.21
Harvesting	OSR	CT	31.690	5.106	127557	0.25	91.5	4575	58357259	233429037	8.37
Harvesting	OSR	CT	33.530	4.552	35611	0.25	96.0	4800	17093394	68373575	7.83
Harvesting	OSR	CT	33.533	4.551	35529	0.25	96.7	4835	17178281	68713125	7.84
Harvesting	OSR	ST	27.713	6.303	2010382	0.25	86.2	4310	866474516	3465898064	9.54
Harvesting	OSR	ST	26.690	6.611	4087481	0.25	88.7	4435	1812797882	7251191529	9.86
Harvesting	OSR	ST	27.617	6.332	2149759	0.25	95.8	4790	1029734449	4118937798	9.61
Harvesting	Wheat	CT	ND	ND	ND	0.25	78.6	3911	ND	ND	ND
Harvesting	Wheat	CT	ND	ND	ND	0.25	76.0	3799	ND	ND	ND
Harvesting	Wheat	CT	ND	ND	ND	0.25	77.9	3887	ND	ND	ND
Harvesting	Wheat	ST	39.703	2.692	493	0.25	80.8	4040	199006	796025	5.90
Harvesting	Wheat	ST	37.533	3.346	2218	0.25	84.3	4215	934930	3739721	6.57
Harvesting	Wheat	ST	31.770	5.082	120673	0.25	76.7	3835	46278271	185113084	8.27
Harvesting	Wheat (C)	CT	47.293	0.407	3	0.25	91.0	4550	1161	4643	3.67
Harvesting	Wheat (C)	CT	47.487	0.349	2	0.25	95.0	4750	1060	4239	3.63
Harvesting	Wheat (C)	CT	44.873	1.136	14	0.25	89.0	4450	6080	24320	4.39
Harvesting	Wheat (C)	ST	35.303	4.018	10412	0.25	83.0	4150	4321108	17284432	7.24
Harvesting	Wheat (C)	ST	36.617	3.622	4188	0.25	86.0	4300	1800962	7203849	6.86

Harvesting	Wheat (C)	ST	37.593	3.328	2128	0.25	81.3	4065	864914	3459658	6.54

Table S7. Ct value and associated calculations for *PhlD* gene copy number per gram of root sample from year 2014/15.

Growth Stage	Crop type	Tillage type	Mean Ct value	Estimated log value	Copy number	Root wt. in g	DNA con. per µl	Total DNA con. in 50 µl	Total copy number	Copy number in g root	Log value g root^{-1}
Vegetative	OSR	CT	33.513	4.557	36025	0.5	118	5900	21254860	42509720	7.63
Vegetative	OSR	CT	30.633	5.424	265411	0.5	108	5400	143322090	286644179	8.46
Vegetative	OSR	CT	30.923	5.337	217063	0.5	112	5600	121555268	243110536	8.39
Vegetative	OSR	ST	27.660	6.319	2086123	0.5	102	5100	1063922649	2127845298	9.33
Vegetative	OSR	ST	27.840	6.265	1841335	0.5	100	5000	920667498	1841334996	9.27
Vegetative	OSR	ST	27.613	6.333	2154783	0.5	110	5500	1185130817	2370261634	9.37
Vegetative	Wheat	CT	35.833	3.858	7210	0.5	146	7300	5263325	10526650	7.02
Vegetative	Wheat	CT	35.260	4.031	10730	0.5	151	7550	8101097	16202194	7.21
Vegetative	Wheat	CT	37.453	3.370	2345	0.5	150	7500	1758470	3516940	6.55
Vegetative	Wheat	ST	31.617	5.128	134211	0.5	130	6500	87237158	174474315	8.24
Vegetative	Wheat	ST	33.733	4.490	30928	0.5	136	6800	21031104	42062207	7.62
Vegetative	Wheat	ST	32.450	4.877	75306	0.5	121	6050	45560120	91120241	7.96
Vegetative	Wheat (C)	CT	33.690	4.503	31872	0.5	107	5350	17051272	34102544	7.53
Vegetative	Wheat (C)	CT	32.223	4.945	88123	0.5	111	5550	48908374	97816749	7.99
Vegetative	Wheat (C)	CT	35.437	3.977	9493	0.5	114	5700	5410887	10821775	7.03
Vegetative	Wheat (C)	ST	31.657	5.116	130540	0.5	105	5250	68533267	137066535	8.14
Vegetative	Wheat (C)	ST	34.520	4.253	17925	0.5	100	5000	8962273	17924546	7.25
Vegetative	Wheat (C)	ST	36.590	3.630	4266	0.5	103	5150	2197223	4394445	6.64
Flowering	OSR	CT	37.447	3.372	2355	0.5	145	7250	1707731	3415461	6.53
Flowering	OSR	CT	36.203	3.747	5578	0.5	142	7100	3960682	7921365	6.90
Flowering	OSR	CT	35.353	4.002	10057	0.5	140	7000	7040241	14080483	7.15
Flowering	OSR	ST	35.547	3.944	8796	0.5	120	6000	5277380	10554760	7.02
Flowering	OSR	ST	34.203	4.349	22326	0.5	113	5650	12614235	25228470	7.40
Flowering	OSR	ST	33.450	4.576	37643	0.5	109	5450	20515192	41030385	7.61
Flowering	Wheat	CT	35.763	3.879	7569	0.5	112	5600	4238438	8476877	6.93
Flowering	Wheat	CT	35.563	3.939	8695	0.5	119	5950	5173267	10346534	7.01
Flowering	Wheat	CT	35.530	3.949	8898	0.5	102	5100	4537916	9075832	6.96
Flowering	Wheat	ST	36.217	3.742	5527	0.5	135	6750	3730784	7461568	6.87
Flowering	Wheat	ST	35.357	4.001	10034	0.5	129	6450	6472103	12944205	7.11
Flowering	Wheat	ST	38.657	3.008	1018	0.5	137	6850	697236	1394472	6.14
Flowering	Wheat (C)	CT	35.580	3.934	8595	0.5	152	7600	6531942	13063885	7.12
Flowering	Wheat (C)	CT	37.323	3.409	2566	0.5	146	7300	1873036	3746072	6.57
Flowering	Wheat (C)	CT	36.657	3.610	4074	0.5	139	6950	2831229	5662458	6.75
Flowering	Wheat (C)	ST	33.543	4.548	35284	0.5	116	5800	20464433	40928866	7.61
Flowering	Wheat (C)	ST	32.617	4.827	67087	0.5	122	6100	40923012	81846024	7.91
Flowering	Wheat (C)	ST	34.307	4.318	20782	0.5	110	5500	11430264	22860529	7.36

Harvesting	OSR	CT	34.427	4.282	19123	0.5	120	6000	11473787	22947574	7.36
Harvesting	OSR	CT	35.287	4.023	10533	0.5	107	5350	5635338	11270676	7.05
Harvesting	OSR	CT	36.520	3.651	4479	0.5	116	5800	2597619	5195237	6.72
Harvesting	OSR	ST	27.633	6.327	2125057	0.5	142	7100	1508790373	3017580745	9.48
Harvesting	OSR	ST	27.797	6.278	1897504	0.5	139	6950	1318765151	2637530303	9.42
Harvesting	OSR	ST	24.823	7.174	14914310	0.5	136	6800	10141730759	20283461518	10.31
Harvesting	Wheat	CT	34.937	4.128	13427	0.5	150	7500	10070015	20140029	7.30
Harvesting	Wheat	CT	36.333	3.707	5098	0.5	155	7750	3950607	7901214	6.90
Harvesting	Wheat	CT	37.650	3.311	2046	0.5	144	7200	1472923	2945845	6.47
Harvesting	Wheat	ST	36.010	3.805	6379	0.5	159	7950	5071089	10142178	7.01
Harvesting	Wheat	ST	36.447	3.673	4712	0.5	153	7650	3604900	7209799	6.86
Harvesting	Wheat	ST	33.343	4.608	40532	0.5	151	7550	30601926	61203851	7.79
Harvesting	Wheat (C)	CT	36.663	3.608	4055	0.5	134	6700	2716798	5433595	6.74
Harvesting	Wheat (C)	CT	38.300	3.115	1303	0.5	133	6650	866805	1733610	6.24
Harvesting	Wheat (C)	CT	35.440	3.976	9471	0.5	138	6900	6534899	13069799	7.12
Harvesting	Wheat (C)	ST	30.630	5.425	266025	0.5	106	5300	140993495	281986990	8.45
Harvesting	Wheat (C)	ST	28.727	5.998	995664	0.5	111	5550	552593502	1105187005	9.04
Harvesting	Wheat (C)	ST	28.963	5.927	844969	0.5	105	5250	443608475	887216949	8.95

Table S8. Ct value and associated calculations for *PhlD* gene copy number per gram of root sample from year 2015/16.

Growth Stage	Crop type	Tillage type	Mean Ct value	Esti-mated log value	Copy number	Root wt. in g	DNA con. per µl	Total DNA con. in 50 µl	Total copy num-ber	Copy num-ber in g root	Log value g root^{-1}
Vegetative	OSR	CT	33.643	4.517	32919.76	0.5	142	7100	23373031	46746062	7.67
Vegetative	OSR	CT	32.427	4.884	76534.34	0.5	140	7000	53574037	107148073	8.03
Vegetative	OSR	CT	31.290	5.226	168330.9	0.5	136	6800	114465039	228930077	8.36
Vegetative	OSR	ST	29.903	5.644	440310.6	0.5	130	6500	286201903	572403805	8.76
Vegetative	OSR	ST	33.510	4.558	36108.55	0.5	135	6750	24373272	48746545	7.69
Vegetative	OSR	ST	33.473	4.569	37038.4	0.5	121	6050	22408234	44816468	7.65
Vegetative	Wheat	CT	35.587	3.932	8555.021	0.5	147	7350	6287941	12575881	7.10
Vegetative	Wheat	CT	34.597	4.230	16996.52	0.5	152	7600	12917354	25834708	7.41
Vegetative	Wheat	CT	36.670	3.606	4036.22	0.5	144	7200	2906078	5812157	6.76
Vegetative	Wheat	ST	32.433	4.882	76181.35	0.5	157	7850	59802359	119604719	8.08
Vegetative	Wheat	ST	31.620	5.127	133901.2	0.5	145	7250	97078336	194156672	8.29
Vegetative	Wheat	ST	33.730	4.491	30999.66	0.5	152	7600	23559744	47119489	7.67
Vegetative	Wheat (C)	CT	33.756	4.484	30445.78	0.5	111	5550	16897407	33794813	7.53
Vegetative	Wheat (C)	CT	34.555	4.243	17494.76	0.5	120	6000	10496854	20993708	7.32
Vegetative	Wheat (C)	CT	32.964	4.722	52727.62	0.5	109	5450	28736554	57473108	7.76
Vegetative	Wheat (C)	ST	34.630	4.220	16608.16	0.5	118	5900	9798817	19597634	7.29
Vegetative	Wheat (C)	ST	33.437	4.580	37992.2	0.5	126	6300	23935085	47870171	7.68
Vegetative	Wheat (C)	ST	30.320	5.518	329822.2	0.5	122	6100	201191516	402383033	8.60
Flowering	OSR	CT	36.643	3.614	4111.549	0.5	146	7300	3001431	6002862	6.78
Flowering	OSR	CT	34.703	4.198	15784.73	0.5	153	7650	12075321	24150643	7.38
Flowering	OSR	CT	34.780	4.175	14967.49	0.5	150	7500	11225620	22451240	7.35
Flowering	OSR	ST	34.510	4.256	18049.27	0.5	143	7150	12905229	25810458	7.41
Flowering	OSR	ST	35.440	3.976	9470.869	0.5	136	6800	6440191	12880381	7.11
Flowering	OSR	ST	31.443	5.180	151351.8	0.5	140	7000	105946251	211892502	8.33
Flowering	Wheat	CT	35.470	3.967	9275.884	0.5	129	6450	5982945	11965890	7.08
Flowering	Wheat	CT	35.600	3.928	8476.289	0.5	133	6650	5636732	11273464	7.05

Flowering	Wheat	CT	35.493	3.960	9127.008	0.5	125	6250	5704380	11408761	7.06
Flowering	Wheat	ST	32.563	4.843	69614.4	0.5	157	7850	54647307	109294615	8.04
Flowering	Wheat	ST	34.697	4.200	15857.87	0.5	153	7650	12131273	24262546	7.38
Flowering	Wheat	ST	35.623	3.921	8340.247	0.5	160	8000	6672197	13344395	7.13
Flowering	Wheat (C)	CT	32.807	4.769	58805.67	0.5	100	5000	29402834	58805667	7.77
Flowering	Wheat (C)	CT	35.480	3.964	9211.785	0.5	141	7050	6494308	12988617	7.11
Flowering	Wheat (C)	CT	33.737	4.489	30856.69	0.5	111	5550	17125462	34250924	7.53
Flowering	Wheat (C)	ST	33.023	4.704	50613.96	0.5	145	7250	36695121	73390241	7.87
Flowering	Wheat (C)	ST	35.443	3.975	9449.003	0.5	139	6950	6567057	13134114	7.12
Flowering	Wheat (C)	ST	33.890	4.443	27744.25	0.5	142	7100	19698414	39396829	7.60
Harvesting	OSR	CT	35.303	4.018	10412.31	0.5	143	7150	7444801	14889601	7.17
Harvesting	OSR	CT	36.550	3.642	4386.447	0.5	138	6900	3026648	6053297	6.78
Harvesting	OSR	CT	34.493	4.261	18259.08	0.5	136	6800	12416173	24832346	7.40
Harvesting	OSR	ST	27.520	6.362	2298799	0.5	133	6650	1528701103	3057402206	9.49
Harvesting	OSR	ST	32.400	4.892	77962.72	0.5	130	6500	50675771	101351542	8.01
Harvesting	OSR	ST	25.767	6.890	7753860	0.5	128	6400	4962470437	9924940875	10.00
Harvesting	Wheat	CT	34.403	4.289	19434.9	0.5	120	6000	11660942	23321884	7.37
Harvesting	Wheat	CT	35.773	3.876	7516.339	0.5	116	5800	4359476	8718953	6.94
Harvesting	Wheat	CT	36.232	3.738	5468.632	0.5	118	5900	3226493	6452986	6.81
Harvesting	Wheat	ST	35.370	3.997	9941.922	0.5	159	7950	7903828	15807655	7.20
Harvesting	Wheat	ST	36.490	3.660	4572.797	0.5	155	7750	3543917	7087835	6.85
Harvesting	Wheat	ST	31.373	5.201	158879.6	0.5	157	7850	124720469	249440939	8.40
Harvesting	Wheat (C)	CT	29.903	5.644	440310.6	0.5	151	7550	332434518	664869035	8.82
Harvesting	Wheat (C)	CT	30.870	5.353	225240.8	0.5	143	7150	161047191	322094382	8.51
Harvesting	Wheat (C)	CT	31.497	5.164	145856.6	0.5	149	7450	108663195	217326390	8.34
Harvesting	Wheat (C)	ST	29.143	5.873	745819	0.5	132	6600	492240559	984481118	8.99
Harvesting	Wheat (C)	ST	35.343	4.006	10127.47	0.5	128	6400	6481582	12963163	7.11
Harvesting	Wheat (C)	ST	27.817	6.272	1871370	0.5	123	6150	1150892515	2301785030	9.36

Calculating copy number (CN) g-1 of soil and g-1 of root samples following qPCR

Sample concentration was determined following amplification cycle. The standard curve was developed in order to calculate the gene CN of 10 μL reaction of each dilution. The mean Ct value of each unknown sample was calculated using the equation line of the standard curve to get the estimated log value. The concentration was then back calculated to determine the CN g-1 of the original soil or root sample. The concentration of each DNA sample (both soil and root) was normalised to 10 ng μL-1, and in each 10 μL reaction mixture 1 μL normalised DNA was used. Further information on these calculations are described here:

Example calculation for soil:

CN g-1 of soil = ((Calculated CN X Original DNA concentration X dilution factor)/10) X 4

DNA concentration used for the reaction

The calculated CN from the Ct value of the sample was 549

Original DNA concentration of the sample was 86 ng μL-1

DNA was dissolved in 50 μL H2O

DNA concentration used for the each reaction was 10 ng μL-1

Soil used for the DNA extraction was 0.25 g

CN g-1 of soil = ((549 X 86 X 50)/10) X 4 = 944,280 copies per g soil

Example calculation for root:

CN g-1 of root = (Calculated CN X Original DNA concentration X dilution factor) X 2

DNA concentration used for the reaction

The calculated CN from the Ct value of the sample was 549

Original DNA concentration of the sample was 108 ng μL-1

DNA was dissolved in 50 μL H2O
DNA concentration used for the each reaction was 10 ng μL-1
Root sample used for the DNA extraction was 0.50 g
CN g-1 of root = (549 X 108 X 50) X 2 = 5929
